# Editorial: Machine intelligence and technology: clinical applications in neurology

**DOI:** 10.3389/fneur.2024.1416270

**Published:** 2024-05-24

**Authors:** Sim Kuan Goh, Jimmy Y. Zhong, Chow-Khuen Chan, Siti Balqis Samdin, Si-Lei Fong, Choong Yi Fong, Kheng Seang Lim

**Affiliations:** ^1^School of Electrical Engineering and Artificial Intelligence, Xiamen University Malaysia, Sepang, Selangor, Malaysia; ^2^Center for Advanced Brain Imaging, Georgia Institute of Technology, Atlanta, GA, United States; ^3^School of Mechanical and Aerospace Engineering, Nanyang Technological University, Singapore, Singapore; ^4^Department of Biomedical Engineering, Faculty of Engineering, Universiti Malaya, Kuala Lumpur, Malaysia; ^5^Department of Biomedical Engineering & Health Sciences, Faculty of Electrical Engineering, Universiti Teknologi Malaysia, Johor Bahru, Malaysia; ^6^Neurology Division, Department of Medicine, Faculty of Medicine, Universiti Malaya, Kuala Lumpur, Malaysia; ^7^Division of Paediatric Neurology, Department of Paediatrics, Faculty of Medicine, Universiti Malaya, Kuala Lumpur, Malaysia

**Keywords:** machine intelligence, neurology, neuroscience, data science, artificial intelligence

Machine intelligence (MI) has emerged as a powerful catalyst reshaping numerous facets of modern society. Key breakthroughs in object detection, content generation, chatbots, robotics, and medical applications have revolutionized how we interact with our surroundings, exchange information, automate tasks, and deliver healthcare services. This transformative success owes much to pivotal enabling technologies such as high-quality sensors and computational hardware capable of simulating complex biological models and training advanced MI algorithms with large amounts of data.

Integrating MI into medical devices and decision support tools holds promising prospects for clinical neurology, especially with wearable sensors and neuroimaging techniques [Functional Magnetic Resonance Imaging (fMRI), Functional Near-Infrared Spectroscopy (fNIRS), Electroencephalography (EEG), Magnetoencephalography (MEG), Computed Tomography (CT), and Positron Emission Tomography (PET)], for diagnosing and treating neurological disorders. However, widespread adoption faces challenges in clinical practice due to the complexity of MI systems, resulting in opaque machine decisions. Negative factors such as uncertainty, bias, unreliability, and violation of privacy posed by MI models can hinder them from being fully trusted by their human users and consequently their ready adoption (1). Moreover, portability, cost, and energy efficiency barriers in current MI and neuroimaging systems limit clinical accessibility, especially for underserved communities. Addressing these challenges is crucial to fully harness MI's potential in enhancing psychological well-being and healthcare outcomes, ensuring equitable access to advanced medical technologies.

Several neurological challenges can be reframed as machine learning problems, presenting opportunities to leverage patient data for data-driven insights. By employing advanced MI algorithms and computational techniques, we can harness the wealth of information embedded within patient records to enhance diagnostic accuracy, tailor treatment approaches, and uncover novel patterns and correlations that may have previously gone unnoticed. This transformative approach holds the potential to revolutionize how neurological disorders are understood and managed. Liu et al. conducted an analysis of the correlation between consciousness states and primary brainstem hemorrhage (PBH). Through the use of Machine learning-based logistic regression, their study showed that (i) general hemorrhage and (ii) hemorrhage specific to the ventricular system were major predictors of their patients' states of consciousness. He et al. proposed the application of machine learning for diagnosing pediatric autism. Utilizing Tract-Based Spatial Statistics derived from diffusion kurtosis imaging, they observed significant alterations in brain microstructure among children with autism compared to standard MRI scans. Additionally, their findings suggested potential neuroimaging biomarkers for pediatric autism diagnosis, including Kurtosis fractional anisotropy (FAK), mean kurtosis (MK), axial kurtosis (KA), and Lateralization index (LI). Wang et al. investigated the effectiveness of artificial intelligence-based CT intracranial hemorrhage detection developed by VeriScout. Their study, conducted amidst influences, such as artifacts and post-operative scans, revealed that the tool surpassed the average sensitivity of radiologists with only a minor trade-off in specificity.

Cheng et al. employed univariable and multivariable linear regression analyses with an intracranial artery feature extraction technique to explore the association of distal arterial morphologic features—including artery length, density, and average tortuosity, measured from 3D Time-of-Flight Magnetic Resonance Angiography (3D-TOF MRA)—with different brain structures such as gray matter volume (GMV), white matter volume (WMV), and cerebrospinal fluid volume (CSFV). Additionally, they investigated correlations between these cerebrovascular characteristics and GMV across different brain regions. Their study improved the methods for evaluating the morphological characteristics of intracranial arteries in Cerebral Small Vessel Disease (CSVD), providing insights to elucidate the relationship between vascular health indicators and brain atrophy.

Computational Fluid Dynamics (CFD) has been as a valuable tool for simulate and analyze fluid flow phenomena, which can be used for understanding of hemodynamics in neurology. Zhang et al. conducted an assessment of the impact of craniocervical junction abnormality (CJA) on vertebral artery hemodynamics. They created three-dimensional reconstruction of the vertebral artery (VA) through the use of head and neck computed tomography angiography (CTA) images, employing CFD techniques. In patients with CJA, notable hemodynamic differences were observed in parameters such as diameter, flow velocity, flow rate, wall pressure, and shear force of the VA compared to the normal population, potentially leading to clinical symptoms such as dizziness. The visualization capabilities of CFD technology facilitate the reconstruction of the vertebral artery in three dimensions, enabling comprehensive analysis of various parameters for pre-operative evaluation and treatment planning.

Wearable sensors integrated with machine intelligence (MI) offer comprehensive and real-time monitoring of neurological development. Ahmed et al. developed and evaluated machine learning models for detecting post-stroke delirium using data from wearable activity monitors and stroke-related clinical features. In their study, patients underwent daily delirium assessments by a neurologist, while wrist-worn actigraph devices recorded activity data throughout hospitalization on both the paretic and non-paretic arms. The analysis revealed the significant contribution of actigraphy data in accurately predicting day-to-day delirium status, with night time actigraphy proving particularly relevant.

Multimodal AI has garnered interest in research and industry due to its capability in integrating diverse sources of information. It has found successful applications in tasks such as image-to-text or text-to-image transformations. In neurology, where multimodal data are routinely available for medical diagnosis and pre-operative planning, exploring multimodal AI is essential. Xia et al. developed a machine learning model that predicts the risk of transient ischemic attack in patients with mild carotid stenosis using radiomics features from CT and clinical information. Their study demonstrated the importance of combining multimodal data. Suh et al. developed and validated a deep learning-based automatic segmentation model for assessing intracranial volume (ICV) and compared the accuracy determined by NeuroQuant, FreeSurfer, and SynthSeg. Specifically, they proposed an ICV segmentation model based on the foundational 2D U-Net architecture trained with four types of input images (both single and multimodality using scaled or unscaled T1-weighted and T2-FLAIR MR). Their model showed promise for accurately evaluating brain atrophy in neurodegenerative disorders, emphasizing the significance of multimodal AI.

The articles within this Research Topic serve to highlight a multitude of fresh and intriguing pathways where machine intelligence (MI) intersects with neurology. Altogether, the studies herein ventured beyond conventional boundaries, leveraging computational models, wearable sensors, and AI frameworks to address neurological diseases. The innovative insights gained from this Research Topic will not only broaden our existing knowledge but shall also pave the way for exciting avenues of future exploration [e.g., Human-AI trust and collaboration (1), AI-driven computational indices of neurocognitive investment (2)].

## Author contributions

SG: Conceptualization, Writing—original draft, Writing—review & editing. JZ: Conceptualization, Writing—review & editing. C-KC: Conceptualization, Writing—review & editing. SS: Conceptualization, Writing—review & editing. S-LF: Conceptualization, Writing—review & editing. CF: Conceptualization, Writing—review & editing. KL: Conceptualization, Supervision, Writing—review & editing.
